# Ocular bacterial infections and antibiotic resistance patterns in patients attending Gondar Teaching Hospital, Northwest Ethiopia

**DOI:** 10.1186/s13104-018-3705-y

**Published:** 2018-08-17

**Authors:** Yeshambel Belyhun, Feleke Moges, Mengistu Endris, Banchamlak Asmare, Bemnet Amare, Damtew Bekele, Solomon Tesfaye, Martha Alemayehu, Fantahun Biadgelegne, Andargachew Mulu, Yared Assefa

**Affiliations:** 1 School of Biomedical and Laboratory Sciences, College of Medicine and Health Sciences, University of Gondar, Gondar, Ethiopia; 2 Arada Sub-city Health Office CDC Project, Addis Ababa City Health Bureau, Addis Ababa, Ethiopia; 3 Department of Biochemistry, College of Medicine and Health Sciences, University of Gondar, Gondar, Ethiopia; 4 Natural and Computational Sciences, Department of Biology, Debre Markos University, Debre Markos, Ethiopia; 5 Natural and Computational Sciences, Department of Biology, University of Gondar, Gondar, Ethiopia; 6 Faculty of Medicine and Health Sciences, Bahir Dar University, Bahir Dar, Ethiopia; 7 Department of Ophthalmology, College of Medicine and Health Sciences, University of Gondar, Gondar, Ethiopia

**Keywords:** Ocular infections, Antibiotic resistance, Ethiopia

## Abstract

**Objectives:**

Empirical selections of antimicrobial therapy based on clinical observations are common clinical practices in Ethiopia. This study identified common external ocular infections and determined antibiotic susceptibility testing in northwest Ethiopia.

**Results:**

Among 210 patients studied, conjunctivitis 32.9%(69), blepharitis 26.7%(56), dacryocystitis 14.8%(51), blepharoconjunctivitis 11.9%(25), and trauma 10.0%(21) were the most common external ocular infections. Pathogenic bacteria were isolated among 62.4%(131) cases. The distributions of bacteria detected in conjunctivitis, dacryocystitis, and blepharitis patients were 32.8%(43), 23.7%(31), and 16.0%(21), respectively. The most prevalent isolates were coagulase negative *Staphylococci*; 27.5%(36), *S. aureus*; 26.7%(35), *Pseudomonas* species; 10.7%(14), and *E. coli*; 7.6%(10). Tetracycline, amoxicillin, chloramphenicol, ampicillin, and nalidic acid showed resistance to bacterial isolates with a respective prevalence of 35.9%(47), 32.1%(42), 26.2%(34), 25.2%(33), and 23.7%(31). Multi-drug resistance patterns to the commonly prescribed antibiotics tested was 20.6%(27), 18.3%(24), 17.6%(23), 5.3%(7), and 4.6%(6) to two, three, four, five, and six antibiotics, respectively. Overall, the multi-drug resistance prevalence rate was 66.4%(87). This study confirmed diverse types of external ocular manifestations associated with bacterial infections with wide ranges of antibiotic resistant phenotypes. Thus, combining clinical information, bacteriological analysis, and antimicrobial susceptibility tests are useful for making an evidence-based selection of antibiotics therapy.

**Electronic supplementary material:**

The online version of this article (10.1186/s13104-018-3705-y) contains supplementary material, which is available to authorized users.

## Introduction

Ocular infections are common in most part of the developing world. In particular, conjunctivitis, keratitis, endophthalmitis, blepharitis, orbital cellulitis, canaliculitis, cellulitis, dacryocystitis, and the likes are common ocular infections caused by bacteria [1, 2]. For effective management of such infections, knowledge of the specific etiology and the structure (s) of the eye affected must be investigated [3]. Moreover, differences in drug absorption, penetration and availability to the structures of the eye, severity of the infections, efficacy and safety of medication, and antibiotic susceptibility pattern could decide for choosing proper antibiotics to each type of ocular bacterial infection [4, 5]. However, treatment for most ocular bacterial infections is primarily empirical with broad-spectrum antibiotics which results in resistance to commonly used antibiotics [2]. Moreover, widespread misuse of broad-spectrum antibiotics such as bacterial, viral infections or prophylactics has resulted in emerging problems of antibiotic resistance [6, 7].

In Ethiopia, eye infections caused by bacteria are important public health problems. Empirical broad-spectrum antibiotics treatments based on clinical observations are routinely practiced. These could be responsible for emerging antibiotic resistance problems over time. We and others reported before [8–10] a high rate of drug resistant bacteria isolates, but without defining the types of ocular manifestations in Ethiopia. However, in studies so far reported in Ethiopia [8, 10–13], the bacterial pathogens and their antibiotic drug resistance profiles were done with no identifications of specific clinical manifestations. As a continuation of our strategy in documenting antibiotic resistance profiles over a period of time [8, 9], in this study, we identified common external ocular infections and determined bacteriological analysis and antimicrobial susceptibility testing.

## Main text

### Materials and methods

#### Sample collection and bacterial identification

A prospective cross-sectional study was conducted among patients who attended the Ophthalmology Department, University of Gondar, Ethiopia. Sociodemographic data collection and relevant clinical evaluations of 210 patients with external ocular infections were done using a structured standard questionnaire. External ocular infections were clinically defined after the patients were examined by an ophthalmologist. Then, eye discharge from each patient was collected using soft-tipped applicators of sterile cotton swabs with due care to avoid possible sources of contamination.

Bacterial identification from eye discharge specimens was performed using standard procedures [14]. Direct Gram staining was done for all specimens. The specimens were inoculated on a proper culture media; blood agar (Oxoid, Hampshire, UK), MacConkey agar (Oxoid, Hampshire, UK) and Chocolate agar (Oxoid, Hampshire, UK) and incubated for 24 h at 37 °C. Bacteria were further characterized and confirmed using colony morphology and by the pattern of their biochemical reactions using the standard biochemical tests procedures, namely, indole production, lactose fermentation, hydrolysis of urea, citrate utilization, lysine decarboxylation, motility test, oxidase test for gram negative bacteria and for gram positive bacteria, mannitol fermentation, and catalase and coagulase tests [14].

#### Susceptibility testing

Once a pure culture was obtained, a loopful of bacteria was taken from the colony and transferred to a tube containing 5 ml tryptone broth and mixed gently until a homogenous suspension was formed. The turbidity of the suspension was adjusted to the optical density of a McFarland 0.5 tube (0.14–0.15 nm) to standardize the inoculums size [15]. The inoculum of each isolate was swabbed onto a Mueller–Hinton (Oxoid, Hampshire, UK), chocolate and blood agars depending on the type of isolated bacteria.

Antimicrobial susceptibility testing was performed using agar disk diffusion technique according to Clinical Laboratory Standard Institute (CLSI) guidelines [16]. The sensitivity/resistance of isolated strains was tested with commonly used antibiotics. The following antibiotic disks were used; ampicillin (10 μg), chloramphenicol (30 μg), gentamicin (10 μg), vancomycin (30 μg), tetracycline (30 μg), co-trimoxazole (25 μg), amoxicillin (20 μg), ciprofloxacin (30 μg), ceftriaxone (30 μg), erythromycin (15 μg), penicillin (10 μg), and methicillin (5 μg) (Oxoid, Hampshire, UK). The antibiotics discs were added after drying the plates for 3–5 min. The plates were incubated aerobically at 37 °C for 24 h. Inhibition zone diameter was measured in millimeter using callipers and was interpreted as susceptible and resistant according to the standardized table supplied by the manufacturers. Reference strains of *S. aureus* (ATCC 25923) and *E. coli* (ATCC25922) were used as controls.

#### Data analysis

Data were entered and analyzed using SPSS version 16 statistical program. Descriptive statistics were used to explain sociodemographic, bacterial prevalence rate and antibiotic resistance/susceptibility patterns.

### Results

#### External ocular infections

Among 210 patients investigated, 60.0%(126) of them were above the age group of 45 years old and 52.3%(110) of them were men. Occupationally, 87.5%(183) of them were farmers and rural dwellers (Additional file 1). The commonest clinical presentation of external ocular infection observed were conjunctivitis; 32.9%(69) followed by blepharitis; 26.7%(56), dacryocystitis; 14.8%(51), blepharoconjunctivitis; 11.9%(25), trauma; 10.0%(21), and others various manifestations; 10.0%(21) (Additional file 1).

#### Bacterial isolates

Among the total of 210 patients, pathogenic bacteria were isolated in 62.4%(131) cases. The most prevalent isolates were coagulase negative *Staphylococcus* (CNS) 27.5%(36), S*. aureus* 26.7%(35), *Pseudomonas* species 10.7%(14), *E. coli* 7.6%(10), and *Klebsiella* species 6.1%(8) (Table 1). Among 131 bacteria; 32.8%(43), 23.7%(31), and 16.0%(21) of the isolates were detected in conjunctivitis, dacryocystitis, and blepharitis, respectively. The 27.5%(36) of bacteria were detected in other ocular infections (Table 1).

**Table 1:** Bacterial isolates in patients with external ocular infections, Gondar University Teaching Hospital, Ethiopia

	Blepharitis	Conjunctivitis	Blepharo-conjunctivitis	External hordeolum	Dacryocystitis	Lid abscesses	Trauma	Others	Total isolates
Gram-positive bacteria									
*S. aureus*	7(20.0)	9(25.7)	5(14.3)	1(2.6)	6(17.1)	1(2.6)	4(11.4)	2(5.7)	35(26.7)
CNS	6(16.6)	9(25.0)	4(11.1)	1(2.8)	9(25.0)	1(2.8)	6(16.7)	–	36(27.5)
*S. pneumoniae*		–	–	–	2(100.0)	–	–	–	2(1.5)
*S. pyogen*	–	1(50.0)	–	–	–	–	1(50.0))	–	2(1.5)
*Enterobacter species*	–	–	1(4.0)	–	2(100.0)	–	–	–	2(1.5)
Gram-negative bacteria									
*E. coli*	1(10.0)	6(60.0)	–	–	1(10.0)	–	1(10.0)	1(10.0)	10(7.6)
*Klebsiella* spp.	–	5(62.5)	1(12.5)	–	2(25.0)	–	–	–	8(6.1)
*Pseudomonas* spp.	1(7.1)	7(50.0)	2(14.3)	–	3(21.4)	–	–	1(7.1)	14(10.7)
*Citrobacter* spp.	–	1(25.0)	1(25.0)	–	1(25.0)	–	–	1(25.0)	4(3.1)
*H. influenzae*	3(30.0)	4(40.0)	–	–	2(20.0)	–	–	1(10.0)	10(7.6)
*P. vulgaris*	1(50.0)	–	–	–	1(50.0)	–	–	–	2(1.5)
*P. mirabilis*	1(33.3)	–	–	–	1(33.3)	–	–	1(33.3)	3(2.3)
*Providencia* spp.	1(33.3)	1(33.3)	–	–	1(33.3)	–	–	–	3(2.3)
Total	21(16.0)	43(32.8)	13(9.9)	2(6.5)	31(23.7)	2(6.5)	12(9.2)	7(5.3)	131(100)

*CNS* coagulase negative *Staphylococci*

#### Antimicrobial susceptibility testing

Overall, 35.9%(47), 32.1%(42), 25.2%(34), 25.2%(33) and 23.7%(31) of the bacterial isolates were resistant to tetracycline, amoxicillin, chloramphenicol, ampicillin, and nalidic acid, respectively. Among major gram positives; *S. aureus* showed 80.0%(28), 91.4%(32), and 94.3%(33) susceptible to ciprofloxacin, methicillin, and ceftriaxone, respectively. Thirty one (86.0%), 88.9%(32), and 91.7%(33) of CNS isolates were susceptible to erythromycin and ceftriaxone, co-trimoxazole, and methicillin, respectively (Table 2).

**Table 2:** Antibiotic resistance pattern of bacterial isolates in external ocular infections, Gondar University Teaching Hospital, Ethiopia

*Bacterial* isolates	No. (%)	Pattern	Common antibiotic tested, No. (%)											
			AMP	AMOX	CIP	CN	MET	NA	CAF	ERY	PEN	TTC	SXT	CEF
*S. aureus*	35(16.7)	S	27(77.1)	26(74.3)	28(80.0)	–	32(91.4)	25(71.4)	23(65.7)	26(74.3)	24(68.6)	22(62.9)	28(63.3)	33(94.3)
		R	8(22.9)	9(25.7)	8(20.09)	–	3(8.6)	10(19.1)	12(34.3)	9(25.7)	11(31.4)	13(16.8)	7(36.4)	2(5.7)
CNS	36(17.1)	S	27(75.0)	26(72.2)	27(77.80)	–	33(91.7)	28(77.8)	28(77.8)	31(86.1)	24(66.7)	26(72.2)	32(88.9)	31(88.6)
		R	9(25.0)	10(27.8)	9(22.2)	–	3(8.3)	8(25.8)	8(25.8)	5(13.9)	12(33.3)	10(27.8)	4(11.1)	4(11.4)
*S. pneumoniae*	2(1.0)	S	0(0)	2(100)	1(50.0)	–	2(100)	2(100)	1(50.0)	2(100)	2(100)	2(100)	2(100)	2(100)
		R	2(100)	0(0)	1(50.0)	–	0(0)	0(0)	1(50.0)	0(0)	0(0)	0(0)	0(0)	0(0)
*S. pyogen*	2(1.0)	S	1(50.0)	2(100)	2(100)	–	2(100)	2(100)	2(100)	1(50.0)	2(100)	1(50.0)	2(100)	1(50.0)
		R	1(50.0)	0(0)	0(0)	–	0(0)	0(0)	0(0)	1(50.0)	0(0)	1(50.0)	0(0)	1(50.0)
*Enterobacter* spp.	2(1.0)	S	2(100)	2(100)	2(100)	–	2(100)	1(50.0)	2(100)	1(50.0)	1(50.0)	2(100)	2(100)	2(100)
		R	0(0)	0(0)	0(0)	–	0(0)	1(50.0)	0(0)	1(50.0)	1(50.0)	0(0)	0(0)	0(0)
*E. coli*	10(4.8)	S	8(80.0)	5(50.0)	8(80.0)	7(70.0)	–	7(70.0)	9(90.0)	–	–	5(50.0)	7(70.0)	9(90.0)
		R	2(20.0)	5(50.0)	2(20.0)	3(30.0)	–	3(30.0)	1(10.0)	–	–	5(50.0)	3(30.0)	1(10.0)
*Klebsiella* spp.	8(3.8)	S	5(62.5)	7(87.5)	5(62.5)	8(100.0)	–	5(62.5)	5(62.5)	–	–	5(62.5)	5(62.5)	7(87.5)
		R	3(37.5)	1(12.5)	3(37.5)	0(0.0)	–	3(37.5)	3(37.5)	–	–	3(37.5)	3(37.5)	1(12.5)
*Pseudomonas* spp.	14(6.7)	S	11(78.6)	7(50.0)	11(78.6)	11(78.6)	–	10(71.4)	10(71.4)	–	–	10(71.4)	9(64.3)	13(92.9)
		R	3(21.4)	7(50.0)	3(21.4)	3(21.4)	–	4(28.6)	4(28.6)	–	–	4(28.6)	5(35.7)	1(7.1)
*Citrobacter* spp.	4(1.9)	S	3(75.0)	1(25.0)	4(100.0)	4(100.0)	–	4(100.0)	3(75.0)	–	–	1(25.0)	4(100.0)	4(100.0)
		R	1(25.0)	3(75.0)	0(0.0)	0(0.0)	–	0(0.0)	1(25.0)	–	–	3(75.0)	0(0.0)	0(0.0)
*H. influenzae*	10(4.8)	S	9(90.0)	6(60.0)	9(90.0)	9(90.0)	–	9(90.0)	8(80.0)	–	–	6(60.0)	8(80.0)	10(100)
		R	1(10.0)	4(40.0)	1(10.0)	1(10.0)	–	1(10.0)	2(20.0)	–	–	4(40.0)	2(20.0)	0(0.0)
*P. vulgaris*	2(1.0)	S	2(100)	1(50.0)	1(50.0)	2(100)	–	2(100)	2(100)	–	–	0(0)	2(100)	1(50.0)
		R	0(0)	1(50.0)	1(50.0)	0(0)	–	0(0)	0(0)	–	–	2(100)	0(0)	1(50.0)
*P. mirabilis*	3(1.4)	S	1(33.3)	2(66.7)	3(100.0)	3(100.0)	–	2(66.7)	1(33.3)	–	–	3(100.0)	3(100.0)	3(100.0)
		R	2(66.7)	1(33.3)	0(100.0)	0(100.0)	–	1(33.3)	2(66.7)	–	–	0(100.0)	0(100.0)	0(100.0)
*Providencia* spp.	3(1.4)	S	2(66.7)	2(66.7)	2(66.7)	3(100.0)	–	2(66.7)	2(66.7)	–	–	1(33.3)	2(66.7)	3(100.0)
		R	1(33.3)	1(33.3)	1(33.3)	0(100.0)	–	1(33.3)	1(33.3)	–	–	2(66.7)	1(33.3)	0(100.0)
Total	131(62.4)	S	98(74.8)	89(67.9)	104(79.4)	47(87.0)	71(92.2)	100(76.3)	96(73.8)	61(78.2)	53(68.8)	84(64.1)	106(80.9)	119(91.5)
		R	33(25.2)	42(32.1)	27(20.6)	7(13.0)	6(7.8)	31(23.7)	34(26.2)	17(21.8)	24(31.2)	47(35.9)	25(19.1)	11(8.5)

*AMP* ampicillin, *AMOX* amoxicillin, *CIP* ciprofloxacin, *CN* gentamycin, *MET* methicillin, *NA* nalidic acid, *CAF* chloramphenicol, *ERY* erythromycin, *VANC* vancomycin, *PEN* penicillin, *TTC* tetracycline, *SXT* co-trimoxazole, *CEF* ceftriaxone

Among gram negatives; *E. coli* showed 50% resistance to amoxicillin and tetracycline and resistance pattern to the rest of antibiotics was equal to or less than 30% (Table 2). Around one-third (37.5%) of *Klebsiella* species were resistant to each of ampicillin, ciprofloxacin, gentamycin, and ceftriaxone. Over 78.6% of *Pseudomonas* species were susceptible to ampicillin, ciprofloxacin, gentamycin, and ceftriaxone. All isolates of *H. influenzae* was sensitive to ceftriaxone, and 90% were also susceptible to ampicillin, ciprofloxacin, gentamycin, and nalidic acid. Antibiotic susceptibility test patterns for the rest of bacterial isolates to each commonly prescribed antimicrobial agent tested are presented in Table 2.

Multi-drug resistance patterns (for two or more antibiotics tested) were common across tested isolates (Table 3). In particular, resistance to six antibiotics was observed in *S. aureus* and CNS. Among common gram negatives; *Pseudomonas* species, *E. coli, Klebsiella* species*, Citrobacter* species and *P. vulgaris* were resistant to four antibiotics (Table 3). Overall; 20.6%(27), 18.3%(24), and 17.6%(23) of bacterial isolates were resistant to two, three, and four antibiotics tested, respectively (Table 3).

**Table 3:** Multiple antibiotic resistance patterns of bacterial isolates in ocular infections, Gondar University Teaching Hospital, Ethiopia

Isolates	No (%)	Antibiotic resistance patterns						
		Ro	R1	R2	R3	R4	R5	R6
*S. aureus*	35(26.7)	5(14.3)	7(20.0)	7(20.0)	4(12.9)	4(12.9)	3(8.6)	5(14.3)
CNS	36(27.5)	8(22.2)	7(19.4)	5(13.9)	3(8.3)	8(22.2)	4(11.1)	1(2.8)
*S. pneumoniae*	2(1.5)		1(50.0)	–	1(50.0)	–	–	–
*S. pyogen*	2(1.5)	1(50.0)	–	–	–	1(50.0)	–	–
*Enterobacter* spp.	2(1.5)		1(50.0)	1(50.0)			–	–
*E. coli*	10(7.6)	1(10.0)	–	3(30.0)	5(50.0)	1(10.0)	–	–
*Klebsiella* spp.	8(6.1)	1(12.5)	2(25.0)	–	2(25.0)	3(37.5)	–	–
*Pseudomonas* spp.	14(10.7)	1(7.1)	3(21.4)	3(21.4)	3(21.4)	4(28.6)		
*Citrobacter* spp.	4(3.1)	1(25.0)		1(25.0)	1(25.0)	1(25.0)	–	–
*H. influenzae*	10(7.6)	1(10.0)	2(20.0)	5(50.0)	2(20.0)	–	–	–
*P. vulgaris*	2(1.5)	–	1(50.0)	–	–	1(50.0)	–	–
*P. mirabilis*	3(2.3)	1(33.3)	–	1(33.3)	1(33.3)	–	–	–
*Providencia* spp.	3(2.3)	–	–	1(33.3)	2(66.7)	–	–	–
Total	131(100)	20(15.3)	24(18.3)	27(20.6)	24(18.3)	23(17.6)	7(5.3)	6(4.6)

R_o_—sensitive to all antibiotics, R_1_—resistant to 1 antibiotic

R_2_—resistant to 2 antibiotics, R_3_—resistant to 3 antibiotics

R_4_—resistant to 4 antibiotics, R_5_—resistant to 5 and more antibiotics

#### Association of ocular infections, bacteria isolates and antibiotic resistance profiles

None of the sociodemographic variables showed significant association with the bacterial isolates and the prevalence of antibiotics resistance. Similarly, the bacterial isolates and the overall antibiotic resistance profiles showed no significant difference with many of the ocular infection types (Additional file 1).

### Discussion

The external ocular bacterial infections identified in this study were diverse in terms of the clinical features, the bacterial isolates, and the antibiotic resistance patterns. Conjunctivitis was the most common ocular infections followed by blepharitis, dacryocystitis, blepharoconjunctivitis, and trauma. The ocular infections types identified in the current study were similar to other parts of Ethiopia [11–13]. A study reported external ocular infections were one of the five top causes of eye morbidity in elderly patients, however, first being cataract, followed by trachoma, presbyopia, and glaucoma [17]. The disparity might be attributed that the latter study was exclusively on elderly patients, but the former studies included all categories of age with external ocular bacterial infections.

About 62.4% pathogenic bacteria were identified in all types of external ocular infections which was higher than our previous retrospective study report of 54.2% [8]. In particular, the bacteria distributions from conjunctivitis and blepharitis patients were significant. The prevalence rate found in the current study was within ranges of the prevalence (from 47 to 74.7%) of bacterial pathogens that were reported in different parts of Ethiopia [8, 10–12, 17]. The most prevalent isolates were CNS, *S. aureus*, *Pseudomonas* species, *E. coli* and *Klebsiella* species [8]. In our previous retrospective study, the commonest isolated bacteria were *S. aureus*, CNS, and *Streptococcus* species [8]. Similarly, in studies so far done in Ethiopia, either *S. aureus or* CNS were the predominant [8–13]. Actually, *S. aureus* was also one of the threats of eye infection and showed a significant increasing trends overtime [18, 19].

In recent times, evolving bacterial resistance represents a worldwide challenge in the management of clinical infections [2, 20] which is also very evident in Ethiopian day-to-day clinical practices [21]. In the current study, tetracycline, amoxicillin, ampicillin, chloramphenicol and nalidic acid were resistant to the bacterial isolates with prevalence rates of 35.9, 32.1, 25.2, 26.2 and 23.7%, respectively. In particular, the potency of ampicillin and tetracycline showed lower efficacy for almost all isolates. Among major gram-positives; *S. aureus* showed 80.0, 88.6, 91.4, and 94.3% susceptibility to ciprofloxacin, vancomycin, methicillin, and ceftriaxone, respectively. Ciprofloxacin susceptibility rate (80.0%) seen in this study was higher than the rate (51.0%) reported before [8, 22]. In line to the current findings, Arantes et al. [23] also reported CNS was the most frequent isolate which showed 70–90% susceptibility rate to vancomycin, chloramphenicol, gentamicin, cefotaxime, oxacillin, ciprofloxacin, and others. In the current study, three isolates each from *S. aureus* and CNS were found to be methicillin resistant. Hospital acquired methicillin resistant *S. aureus* (MRSA) has been involved with ocular infections in association to hospital acquired exposure [3] and thus need a special emphasis to avoid emergence and spread of MRSA.

Among gram-negatives; *E. coli* showed 50% resistance to amoxicillin and tetracycline, and resistance pattern to the rest of antibiotics were less than 31%. Around one-third of *Klebsiella* species were resistant to ampicillin, ciprofloxacin, gentamycin, and ceftriaxone. The resistance to *Pseudomonas* species to ampicillin, ciprofloxacin, gentamycin, and ceftriaxone seek great attention. Of course, a similar higher incidence resistance rate to commonly used antibiotics was found for most gram-negative bacteria, particularly *Pseudomonas* species [3]. Gram-negative bacteria, mainly *Pseudomonas*, were the most common multidrug-resistant bacteria [19]. Nevertheless, we found that all isolates of *H. influenzae* were susceptible to ceftriaxone and 90% of it also susceptible to ampicillin, ciprofloxacin, gentamycin and nalidic acid.

The emergence of multidrug resistant strains of bacteria is quite challenging [20] and becomes a matter of concern globally [2, 20, 21]. In the current study, most bacterial isolates showed resistance pattern to more than two antibiotics with the overall prevalence of 66.4%, but less than our retrospective study (77.3%) [8] and earlier report (87.1%) of the area [10]. Specific to the dominant bacterial species identified, for instance, we found a higher rate of multi-drug resistant isolates (to six antibiotics) for *S. aureus* and CNS. In addition, many of the isolates such as *Pseudomonas* species, *E. coli*, *Klebsiella* species*, Citrobacter* species and *P. vulgaris* were resistant to four antibiotics tested. Overall, 20.6%, 18.3%, and 17.6% of bacterial isolates were resistant to two, three, and four commonly prescribed antibiotics tested in this study.

In addition to the obvious selective pressures for an antimicrobial drug resistance mechanisms [7, 8], the frequent usage of the antibiotics by the patients before visiting the hospital could be responsible for the high percentage of resistance pattern in this study. For instance, 28.6% of the attendants had a history of previous antibiotics use without physician prescription before their hospital visit. However, but none of the sociodemographic variables showed significant association with the bacterial isolates and the overall prevalence of antibiotics resistance rate. Moreover, no certain types of eye infections were more prone to the overall prevalence of the antibiotic resistance rate.

### Conclusions

The clinical information, bacteriological analysis and associated antimicrobial susceptibility rate observed in this study are useful for making an evidence-based selection of antibiotics therapy in the setting. We recommend a similar study over a period of time to follow antibiotic susceptibility patterns.

## Limitations

Facility and resource limitations hindered similar characterization of ocular external infections from anaerobic bacteria, fungal and viral pathogens.

## Additional file

**Additional file 1.** Comparison of the distribution of the sociodemographic and external ocular infections with total bacteria isolates and associated antibiotic resistance profiles.

## Abbreviations

CNS: coagulase negative *Staphylococcus*
MRSA: methicillin resistant *Staphylococcus aureus*
