# Supplementary material for: Ocular bacterial infections and antibiotic resistance patterns in patients attending Gondar Teaching Hospital, Northwest Ethiopia
Source: BMC Res Notes. 2018 Aug 17;11:597. doi: 10.1186/s13104-018-3705-y (PMC6098601; doi:10.1186/s13104-018-3705-y)
Supplement: Supplementary file 1 — Additional file 1. Comparison of the distribution of the sociodemographic and external ocular infections with total bacteria isolates and associated antibiotic resistance profiles. [file 13104_2018_3705_MOESM1_ESM.docx]

Additional file 1. Distribution of sociodemographic and external ocular infections with total bacteria isolates and antibiotic resistance profiles.

|  | **Frequency, n (%)** | | | | | | | |
| --- | --- | --- | --- | --- | --- | --- | --- | --- |
|  | **Bacterial isolates** | | **X²** | **P-value** | **Resistance to any antibiotic tested** | |  | **P-value** |
|  | **detected** | **Not detected** |  |  | **Yes** | **No** | **X²** |  |
| **Sociodemographic variables:** |  |  |  |  |  |  |  |  |
| **Sex:** Female | 68 (68.0) | 32(32.0) | 2.7 | 0.10 | 57(83.8) | 11(16.2) | 0.81 | 0.67 |
| Male | 62(56,9) | 47(43,1) |  |  | 55(88.7) | 7(11.3) |  |  |
| **Age in years:** ≤ 15 | 22(62.9) | 13(37.1) | 4.4 | 0.36 | 21(95.5) | 1(4.5) | 2.8 | 0.09 |
| 16-30 | 11(68.8) | 5(31.2) |  |  | 10(90.9) | 1(9.1) |  |  |
| 31-45 | 14(46.7) | 16(53.3) |  |  | 12(85.7) | 2(14.3) |  |  |
| 46-60 | 42(66.7) | 21(33.3) |  |  | 34(81.0) | 8(19.0) |  |  |
| > 61 | 42(66.7) | 21(33.3) |  |  | 36(85.7) | 6(14.3) |  |  |
| **Patients’ address** |  |  |  |  |  |  |  |  |
| Gondar city & its vicinity | 59(69.4) | 26(30.6) | 9.4 | 0.31 | 53(89.8) | 6(10.2) | 7.5 | 0.49 |
| Dembia-Takusa-Alefa route | 12(50.0) | 12(50.0) |  |  | 10(83.3) | 2(16.7) |  |  |
| Chilga-Metema route | 6(40.0) | 4(60.0) |  |  | 4(66.7) | 2(33.3) |  |  |
| Tikildingay-Dansha-Humera route | 4(50.0) | 4(50.0) |  |  | 4(100.0) | 0(0.0) |  |  |
| Wegera-Dabat-Debark route | 7(77.8) | 2(22.8) |  |  | 5(100.0) | 0(0.0) |  |  |
| Debretabor town & its vicinity | 11(57.9) | 8(42.1) |  |  | 5(71.4) | 2(28.6) |  |  |
| Others | 23(65.7) | 12(34.3) |  |  | 18(78.3) | 5(21.7) |  |  |
| **Occupation** |  |  |  |  |  |  |  |  |
| Agricola based | 111(60.7) | 72(39.3) | 1.8 | 0.18 | 95(85.6) | 16(14.4) | 0.28 | 0.60 |
| Non-agriculture based | 20(74.1) | 7(25.9) |  |  | 18(90.0) | 2(10.0) |  |  |
| **History of other chronic illness** |  |  |  |  |  |  |  |  |
| Yes | 1(50) | 1(50.0) | 0.13 | 0.72 | 0(0.0) | 1(100.0) | 6.3 | 0.12 |
| No | 130(62.5) | 78(37.5) |  |  | 113(86.9) | 17(13.1) |  |  |
| **Previous antibiotic use** |  |  |  |  |  |  |  |  |
| Yes | 39(66.1) | 20(33.9) | 1.2 | 0.55 | 36(92.3) | 3(7.7) | 2.0 | 0.37 |
| No | 90(60.4) | 59(39.6) |  |  | 75(83.3) | 15(16.7) |  |  |
| I don’t know | 1(100) | 0(0.0) |  |  | 1(100) | 0(0.0) |  |  |
| **Duration of symptoms** |  |  |  |  |  |  |  |  |
| Weeks | 15(68.2) | 7(31.8) | 1.9 | 0.60 | 13(86.7) | 2(13.3) | 1.1 | 0.79 |
| Months | 20(71.4) | 8(28.6) |  |  | 18(90.0) | 2(10.0) |  |  |
| Years | 30(62.5) | 18(37.5) |  |  | 27(90.0) | 3(10.0) |  |  |
| I don’t know | 66(58.9) | 46(41.1) |  |  | 55(83.3) | 11(16.7) |  |  |
| **External ocular infections types** |  |  |  |  |  |  |  |  |
| **Blepharitis**: Yes | 28(50.0) | 28(50.0) | 5.0 | 0.03 | 23(81.1) | 5(17.9) | 0.51 | 0.48 |
| No | 103(66.9) | 51(33.1) |  |  | 90(87.4) | 13(12.6) |  |  |
| **Conjunctiviti**s |  |  |  |  |  |  |  |  |
| Yes | 53(76.8) | 16(23.2) | 9.1 | 0.003 | 45(84.9) | 8(15.5) | 0.14 | 0.70 |
| No | 78(55.3) | 63(44.7) |  |  | 68(87.2) | 10(12.8) |  |  |
| **Blepharoconjunctivitis** |  |  |  |  |  |  |  |  |
| Yes | 20(80.0) | 5(20.0) | 3.8 | 0.08 | 18(80.0) | 2(20.0) | 0.28 | 0.60 |
| No | 111(60.0) | 74(40.0) |  |  | 95(85.6) | 16(14.4) |  |  |
| **External hordeolum** |  |  |  |  |  |  |  |  |
| Yes | 2(100.0) | 0(0.0) | 0.14 | 0.53 | 2(100.0) | 0(0.0) | 0.32 | 0.57 |
| No | 129(62.0) | 79(38.0) |  |  | 111(86.0) | 18(14.0) |  |  |
| **Dacryocystitis** |  |  |  |  |  |  |  |  |
| Yes | 30(60.0) | 20(40.0) | 0.16 | 0.69 | 27(90.0) | 3(10.0) | 0.46 | 0.50 |
| No | 101(63.1) | 59(36.9) |  |  | 86(85.1) | 15(14.9) |  |  |
| **Lid abscesses** |  |  |  |  |  |  |  |  |
| Yes | 2(66.7) | 1(33.3) | 0.24 | 1.00 | 2(100.0) | 0(0.0) | 0.32 | 0.57 |
| No | 129(62.3) | 78(37.7) |  |  | 111(86.0) | 18(14.0) |  |  |
| **Trauma** |  |  |  |  |  |  |  |  |
| Yes | 10(47.6) | 11(52.4) | 2.2 | 0.16 | 8(80.0) | 2(20.0) | 0.36 | 0.55 |
| No | 121(64.0) | 68(36.0) |  |  | 105(86.8) | 16(13.2) |  |  |
| **Others*** |  |  |  |  |  |  |  |  |
| Yes | 12(57.1) | 9(42.9) | 0.3 | 0.64 | 12(100.0) | 0(0.0) | 2.10 | 0.15 |
| No | 119(63.0) | 70(37.0) |  |  | 101(84.9) | 18(15.0) |  |  |

*Vernal keratoconjunctivitis, painful blind eye, post traumatic endophthalmitis, post cataract extraction, keratitis, trachoma, endaptrmarty, carcinoma, corneal discharge, chronic anterior uveitis, stromal abscess.
